# The efficacy of inactivated West Nile vaccine (WN-VAX) in mice and monkeys

**DOI:** 10.1186/s12985-015-0282-8

**Published:** 2015-04-09

**Authors:** Yuko Muraki, Takeshi Fujita, Masaaki Matsuura, Isao Fuke, Sadao Manabe, Toyokazu Ishikawa, Yoshinobu Okuno, Kouichi Morita

**Affiliations:** Kanonji Institute, The Research Foundation for Microbial diseases of Osaka University, Yahata-cho 2-9-41, Kannonnji City, Kagawa 768-0061 Japan; Department of Virology, Institute of Tropical Medicine, Nagasaki University, Sakamoto-machi 1-12-4, Nagasaki City, Nagasaki 852-8523 Japan

**Keywords:** West Nile Virus, Formalin-inactivated vaccine, Challenge test in mouse, Efficacy in monkey

## Abstract

**Background:**

West Nile virus (WNV) belonging to the genus *Flavivirus* of the family *Flaviviridae* causes nervous system disorder in humans, horses and birds. Licensed WNV vaccines are available for use in horses but not for humans. We previously developed an inactivated West Nile virus vaccine (WN-VAX) using a seed virus from West Nile virus (WNV NY99) that was originally isolated in New York City in 1999. In this study, we report the immunogenicity of WN-VAX in both mice and non-human primates.

**Findings:**

The WN-VAX immunized mice showed protection against lethal infection with WNV NY99. The challenge test performed on mice passively immunized with serum from other mice that were previously immunized with WN-VAX confirmed that the neutralizing antibody titers of more than 1log10 protected the passively immunized mice from WNV lethal infection. Furthermore, monkeys (*Macaca fascicularis*) immunized three times with 2.5 μg, 5 μg or 10 μg/dose of WN-VAX exhibited neutralizing antibodies in their sera with titers of more than 2log10 after the second immunization.

**Conclusions:**

The WN-VAX was protective in mice both by active and passive immunizations and was immunogenic in monkeys. These results suggest that the vaccine developed in this study may be a potential WNV vaccine candidate for human use.

## Findings

### Background

West Nile virus (WNV) belonging to the genus *Flavivirus* of the family *Flaviviridae* has caused sporadic disease epidemics in Africa, Europe, Middle East and West Asia. Until the end of the 1990s, WNV disease was not taken seriously because it was believed to be a mild febrile infection. Later, this virus was found to be highly pathogenic to humans, horses and birds. A strain of this virus that spread in New York over a short period of time was isolated [1]. This strain has caused high rates of nervous system disorder and mortality, particularly in the elderly population [2].

There are commercially available licensed WNV vaccines for horses, but there are currently none available for humans. Candidate vaccines, such as the chimera vaccine with YFV, inactivated vaccine and DNA vaccine, for human use are still under development (Table 1) [3-6].

**Table 1:** **WNV vaccine for horse and candidate WNV vaccines for humans**

**Compared features**	**WN-VAX**	**Duvaxyn WNV vaccine**	**Candidate vaccine from the Indian group** **[** 3 **]**	**VRC** **[** 4 **]**	**ChimeriVax-WN02** **[** 5 **]**	**WN/DEN4Δ30** **[** 6 **]**
Type	Inactivated	Inactivated	Inactivated	DNA-vectored vaccine	Live, attenuated chimeric vaccine	Live, attenuated chimeric vaccine
Immunogen	Whole virion (NY99)	Whole virion VM-2	Whole virion WNIRGC07	WNV prM/E (NY99) Vector: VR-1012 (CMV/R backbone).	WNV prM/E (NY99) Backbone: Yellow Fever vaccine 17D	WNV prM/E Backbone: dengue virus type 4
Method of inactivation	Formalin treatment	Formalin treatment	Formalin treatment	NA	NA	NA
Adjuvant	None	Squalane	unknown	NA	NA	NA
		Pluronic L121				
		Polysorbate 80				
Preservative	None	Thimerosal	unknown	NA	NA	NA
Target species	human	horse	human	human	human	human
Stage of clinical trial	Pre -clinical	Commercial vaccine	Pre-clinical	Phase 1	Phase II	Phase 1

NA: not applicable.

We previously reported the development of an inactivated and preservative-free WNV vaccine (WN-VAX) for human use. The method used for the production of this vaccine candidate was similar to that used to produce the cell-culture-derived inactivated Japanese encephalitis (JE) vaccine [7,8]. In this study, we report the immunogenicity of WN-VAX in both mice and non-human primates.

## Materials and methods

### Inactivated West Nile vaccine and neutralizing antibody titer (NAT) determination

The WNV strain (NY99-35262-11), which was isolated from a flamingo in New York in 1999, was used to prepare WN-VAX in Vero cells [8]. The ddY mice and the monkeys (*Macaca fascicularis*) used in this study were obtained from Japan SLC, Shizuoka, Japan. The NATs of the serum of immunized animals against WNV were determined following a procedure developed for JE virus with some modifications [9,10].

### Challenge of WN-VAX immunized mice

Four-week-old female mice were divided into five groups (n = 10 per group). All of the members of each group were immunized subcutaneously with 0.5 ml of WN-VAX at a specific concentration from a four-fold dilution series (0.313, 0.078, 0.02, 0.005 and 0.001 μg/dose) with phosphate-buffered saline not containing calcium and magnesium but containing 0.02% gelatin as the diluent. A control group of 10 mice was left untreated. Immunization was performed twice with a seven-day interval. Seven days after the second immunization, both the immunized and the non-immunized mice were challenged intraperitoneally with 2.7 × 10^7^ PFU of WNV NY99 per mouse (10^8.46^ LD50). The survival of the mice was observed for 21 days, and the survival rate per group was computed.

### Challenge and NATs of passively immunized mice

To obtain antisera, four-week-old female mice were immunized twice with 5 μg of WN-VAX at seven-day interval. One week after the second immunization, serum samples were collected, pooled (1:1) and diluted in a four-fold series (1:4, 1:16, and 1:64). For passive immunization, 0.5 ml of undiluted or diluted sample was administered subcutaneously into each member of four groups (n = 15 per group) of six-week-old female mice. A control group of 10 mice was not immunized. After 24 hours, 10 out of 15 passively immunized mice per group and all of the control mice were challenged with 6.4 × 10^3^ PFU of WNV NY99 per mouse (10^3.65^ LD50), whereas serum samples from the five remaining unchallenged passively immunized mice from each group were collected for the determination of NATs against WNV NY99. The NATs for the serum samples from the mice that served as sources of passive immunization were also determined. The mortality of the challenged mice was then observed over a period of 21 days.

### Immunogenicity of WN-VAX in monkeys

Ten female five-year-old monkeys were divided into three groups (n1 = 4, n2 = 3, n3 = 3), and each group was immunized subcutaneously with 2.5 μg, 5 μg, and 10 μg per dose, respectively. The monkeys were immunized on day 0 and on days 14, and 35. Serum from each monkey was collected before immunization and 7, 14, 21, 28, 35, 42, 49, and 56 days after the first immunization and checked for NATs against WNV NY99.

### Statistical analysis

The murine survival curves were compared with the challenge group using the logrank test. Differences were considered statistically significant when P <0.05 by the Dunnett-Hsu multiple comparison test. In monkeys, statistical analysis was performed using Microsoft Excel 2000, and the P values were calculated through the paired Student’s *t*-test.

### Ethical approval

The experiments were performed in accordance with internal procedures and were approved by the Institutional Animal Care and Use Committee at the Research Foundation for Microbial Diseases of Osaka University.

## Results

### Protective efficacy of WN-VAX in mice

**Figure 1:**
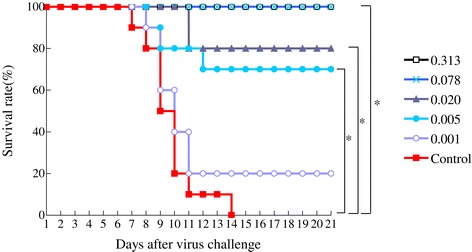
**Survival rate of WN-VAX immunized mice and control.** Five groups of mice (n = 10/group) were immunized twice with 0.313 (□/black), 0.078 (x/pale blue), 0.02 (▲/blue gray), 0.005 (●/sky blue) and 0.001 (○/light purple) μg/dose of WN-VAX at an interval of seven days. The control group (■/red, n = 10) was left untreated. Seven days after the second immunization, both the immunized and the control groups of mice were challenged with WNV NY99, and their survival was observed for 21 days thereafter. The survival rate increased correspondingly with an increase in the concentration of WN-VAX. P values were calculated using the Dunnett-Hsu multiple comparison test. The arithmetic means of survival time for the groups of mice immunized with 0.313, 0.078, 0.02, 0.005 and 0.001 μg/dose of WN-VAX were 21.0., 21.0, 18.8, 17.3 and 11.1 days. *P <0.05.

Mice immunized with WN-VAX were protected from lethal challenge with WNV NY99. The survival rate was correlated with the immunization dose of WN-VAX. The survival rate was 100% at a concentration of WN-VAX of at least 0.078 μg/dose (Figure 1). We observed no clinical signs in the surviving mice. None of the non-immunized mice survived the challenge.

### Protective efficacy of WN-VAX induced neutralizing antibody in passively immunized mice

**Figure 2:**
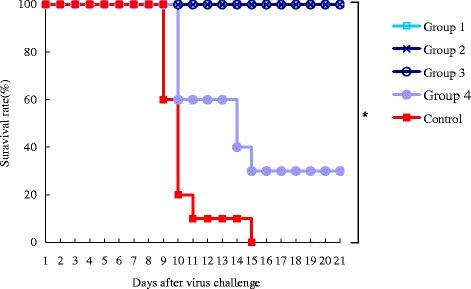
**Survival rates of passively immunized mice and control.** Four groups of mice (n = 10/group) were passively immunized with serum with NAT titers of 2.62 (□/sky blue, group 1), 2.09 (x/dark blue, group 2), 1.65 (○/navy blue, group 3) and 1.01 log10 (●/pale blue, group 4). The NATs from the passively immunized mice are shown in Table 2. The control group (■/red, n = 10) was left untreated. Twenty-four hours after serum administration, both the immunized and the control groups of mice were challenged with WNV NY99, and their survival was observed for 21 days thereafter. All of the passively immunized mice in groups 1, 2 and 3 survived. P values were calculated using Dunnett-Hsu multiple comparison test. *P <0.05.

The NATs in the undiluted (1:1) and the serially diluted (1:4, 1:16 and 1:64) serum samples of WN-VAX immunized mice were 2.62, 2.09, 1.65 and 1.01log10, respectively. The survival rates of the groups of mice (Groups 1, 2, 3 and 4) passively immunized with these immune sera and infected with lethal WNV 24 hours post-immunization were 100% in the first three groups and 30% in the last group. In Group 3, only one mouse was NAT-positive against WNV, and the rest were negative (under the detection limit). None of the control mice survived after lethal infection with WNV (Table 2, Figure 2).

**Table 2:** **Neutralizing antibody titers (NATs) and survival rates in mice**

**Group**	**NATs in pooled serum** ^**a**^ **of WN-VAX immunized mice (serum dilution)**	**NATs in serum of of individual mouse** ^**b**^ **24 hrs after passive immunization**	**Survival rate of passively immunized mice** ^**c**^ **after challenge with WNV NY99**
1	2.62^d^	1.72^d^	100% (10/10)^e^
	(1:1)	1.66	
		1.73	
		1.73	
		1.93	
2	2.09	1.55	100% (10/10)
	(1:4)	1.36	
		1.23	
		1.33	
		1.27	
3	1.65	1.03	100% (10/10)
	(1:16)	<1.00	
		<1.00	
		<1.00	
		<1.00	
4	1.01	<1.00	30% (3/10)
	(1:64)	<1.00	
		<1.00	
		<1.00	
		<1.00	
Control		Not tested	0% (0/10)

^a^used for passive immunization of mice in Groups 1, 2, 3 and 4.

^b^Only 5 mice per experimental group had their serum determined for NATs. These mice were not subjected to virus challenge and were used only for a representative determination of NATs in the group.

^c^NATs of their serum were not determined because no blood sampling was done to avoid stressing the animals during virus challenge.

^d^NAT log10.

^e^number enclosed in parentheses refer to the number of surviving mice/total no. of mice.

### Immunogenicity of WN-VAX in monkeys

**Figure 3:**
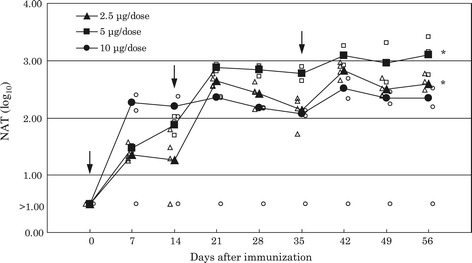
**Neutralization titers in the groups of monkeys immunized with WN-VAX.** Geometric mean neutralization titers (GMT) in the groups of monkeys immunized with 2.5 μg/dose (▲), 5 μg/dose (■) or 10 μg/dose (●) of WN-VAX. NATs from the serum of each monkey immunized with 2.5 μg/dose (∆), 5 μg/dose (□) or 10 μg/dose (○). In the groups that received 5 μg/dose and 10 μg/dose WN-VAX, the NATs reached a plateau after the second immunization. The arrow points to the day of WN-VAX immunization. P values were calculated using the paired Student’s t-test against the NAT from the monkeys at day 0 before immunization. *P <0.05.

All of the immunized monkeys, with the exception of one (non-responder) belonging to the group administered 10 μg/dose of WN-VAX, had more than 1log10 NATs after receiving a single dose of WN-VAX. All of the monkeys except the non-responder had more than 2log10 NATs after the second immunization. The geometric mean titers (GMTs) were calculated using the data from seropositive monkeys in each group. The GMTs reached a plateau at a value of more than 2log10 after the monkeys received the vaccine for the second time (Figure 3).

## Discussion

Our previous study showed that the WN-VAX developed by our laboratory is immunogenic in mice, is capable of eliciting neutralizing antibodies in mice and can protect WNV-immunized mice from lethal WNV infection [8]. Here, we showed that the vaccine is protective in mice through not only active but also passive immunization and is also immunogenic in monkeys.

A previous study reported that hamsters passively immunized with the sera from other hamsters receiving the inactivated WNV vaccine for horses were protected from lethal infection with WNV [11]. In our study, we applied the 50% plaque reduction assay for measuring the NATs against WNV NY99. This method, which is recommended by the WHO for testing the potency of the JE vaccine, has a cut-off value of 1log10 for seroprotection [10]. We noted that the NATs in the serum of one mouse from a group (Group 3, Table 2) that received the WN-VAX immune serum at a NAT of 1.65log10 were 1.03log10, and the corresponding values in the other groups were less than that recommended for seroprotection against JEV [10,12]. Despite the low NATs, all of the mice in Group 3 survived the challenge, which suggests that WNV lethal infection is preventable in mice as long as the antiserum used for passive immunization has a NAT of more than 1log10. It has been clinically shown that the inoculation of immunoglobulins containing high titers of antibodies against WNV has therapeutic effects on patients with neurological disorder caused by WNV infection [13-15]. However, there are no reports on the use of these antibodies for preventive purposes in humans.

WNV, similarly to JEV, belongs to the JE serocomplex. The WN-VAX in this study was produced based on the procedure for the production of inactivated JE vaccine (JEBIK V® The Research Foundation for Microbial Diseases of Osaka University). Epidemiological evidence in a JE-endemic area suggests that the JE vaccine may be effective for protecting people from JE disease, and the challenge test with the inactivated JE vaccine demonstrated that NATs of more than 1log10 can prevent mice from the onset of illness [12]. NATs of more than 1log10 were also found to be effective for protecting humans from JEV infection [16]. The protective mechanism provided by WN-VAX to an organism is supposed to be similar to that of JEV, and the present study demonstrated that the actively and the passively immunized mice were protected from WNV lethal infection. Thus, it is also possible that NATs of more than 1log10 can protect human beings from WNV lethal infection.

In monkeys, the NATs against WNV increased more than 2log10 after at least two subcutaneous immunizations of WN-VAX, regardless of the immunization dose (2.5 μg, 5 μg or 10 μg/dose). Because NATs of 1log10 protected mice from WNV lethal infection, WN-VAX may also protect monkeys from this lethal infection at the same NAT level. Given that the NATs against WNV in monkeys with a dose of more than 5 μg/dose reached a plateau after the second immunization, it is considered that, at least, a certain level of protective ability against WNV infection was acquired three weeks after the first immunization. Thus, three doses enabled those immunized monkeys to maintain the level of NATs required for protection from infection. Protection experiments in monkeys are now considered for investigation.

While most cases of WNV infections are subclinical, the elderly population shows a high incidence of serious nervous disorders and thus a safe WNV vaccine is needed. Our GLP test on WN-VAX indicates no safety problems [8]. Clinical trials on the safety and efficacy of WN-VAX on humans will be performed in our future research.
